# Exosomal microRNAs Targeting *TP53* Gene as Promising Prognostic Markers for Head and Neck Squamous Cell Carcinoma

**DOI:** 10.1055/s-0042-1758204

**Published:** 2022-12-14

**Authors:** Vijayashree Priyadharsini Jayaseelan, Paramasivam Arumugam

**Affiliations:** 1 Clinical Genetics Lab, Centre for Cellular and Molecular Research, Saveetha Institute of Medical and Technical Sciences (SIMATS), Saveetha University, Saveetha Dental College and Hospital, Chennai, Tamil Nadu, India; 2 Molecular Biology Lab, Centre for Cellular and Molecular Research, Saveetha Institute of Medical and Technical Sciences (SIMATS), Saveetha University, Saveetha Dental College and Hospital, Chennai, Tamil Nadu, India

**Keywords:** microRNAs, exosomes, *TP53*, HNSCC, theragnostic

## Abstract

**Statement of Problem** MicroRNAs are small non-coding RNAs that regulate an array of functions by targeting crucial genes. A significant dysregulation in the *TP53* profile has been observed in the head and neck squamous cell carcinoma (HNSCC) patients. Hence, the present *in silico* study was designed to identify those microRNAs which target *TP53* gene and demonstrate their differential expression in HNSCC cases.

**Materials and Methods** The study was extended further to explore their exosomal location using database such as EVmiRNA and ExoCarta. The study follows an observational *in silico* design. Computational tool miRDB was used identify the microRNA targets of *TP53* gene. The UALCAN server was used to ascertain the expression of microRNA in HNSCC cases derived from the Cancer Gene Atlas dataset. The survival of HNSCC patients based on the differential expression microRNA markers were recorded. Further, each of the microRNA was queried for their exosomal presence using EVmiRNA.

**Results** About 102 microRNA targets of *TP53* gene with a target score in the range of 95–50 were identified. The differential expression data for 52 microRNAs was retrieved from the UALCAN database. The microRNAs hsa-miR-421, hsa-miR-548f-5p, and hsa-let-7c-5p were found to be differentially expressed with marked influence over the survival of HNSCC patients. Furthermore, hsa-miR-421 and hsa-let-7c-5p were found to have an exosomal origin especially in body fluids such as blood and saliva.

**Conclusion** The results accumulated from the present study identified three microRNAs which can affect the functions of *TP53* gene and bring about serious outcomes in HNSCC patients. The microRNAs of exosomal origin targeting *TP53* gene in HNSCC patients can be a promising prognostic marker, which can be further used as a therapeutic lead by designing inhibitors.

## Introduction

Head and neck squamous cell carcinoma (SCC) is considered to be the most common type of cancer afflicting individuals predominantly from the South Asian region. The incidence rate of lip and oral cavity cancer has been recorded as 10.2 per 100, 000 contributed by the steeply increasing number of cases in the Indian subcontinent. 1 Several factors have been attributed to the development of cancer such as chronic exposure to substances, oral hygiene, inflammatory diseases, sharp tooth, etc., 2 Alongside, the genetic basis of oral cancer has been dissected and numerous genetic associations have been established which directly or indirectly precipitate the disease phenotype. One among them is the *TP53* gene (tumor suppressor protein), a key regulator of apoptosis, cell cycle regulation, and DNA repair process. Innumerable studies have focused on the role of *TP53* gene in various cancer types including oral SCC. A very recent study investigated the mutation profile of metastatic head and neck cancer in comparison to primary tumors. Interestingly, the team observed that the mutation frequency was lower in metastatic tumors with an enrichment of missense mutations at the DNA binding region of the *TP53* gene. Therefore, it is clear that *TP53* mutations have a greater influence over clinical and molecular aspects of tumorigenesis pertaining to metastasis and therapeutic response. 3

Although genetic alterations encompassing the proto-oncogenes, oncogenes, and tumor suppressor gene, are common observations in cancer, epigenetic modifications also play a very vital role in the disease process. The DNA methylation, histone modifications, and non-coding RNAs have been implicated in the development, progression, and aggressiveness of cancer. Among all the epigenetic marks, the non-coding regulatory molecules such as the miRNAs target multiple genes and modulate their expression. 4 The miRNAs targeting *TP53* gene could influence the gene expression process eventually leading to dysfunctioning of the *TP53* cascade. Hence, a comprehensive understanding about the miRNAs targeting *TP53* gene could aid in designing therapeutic lead of inhibitors against microRNA, thereby restoring the functions of *TP53* gene. Also, the presence of differentially expressed miRNAs (DEMs) targeting *TP53* can be used as promising prognostic markers. These markers are encapsulated within vesicular bodies called exosomes and secreted into the body fluids. 5 Recovering these DEMs from exosomes could offer a less invasive mode of diagnosis of head and neck squamous cell carcinoma (HNSCC) patients at different stages of tumor development. The prognostic ability of these markers can be further validated using more precise experimental designs.

## Material and Methods

### Study Subjects

The cBioPortal (The cBio Cancer Genomics Portal) database is an interactive pipeline for analyzing cancer datasets. It provides access to 5,000 tumor samples for nearly 20 different studies. It provides an intuitive way to gain access to clinical attributes and molecular profiles of cancer patients. This portal ( http://cbioportal.org ) was used in the present study to acquire information about the *TP53* mutation status in HNSCC patients. TCGA (The Cancer Gene Atlas) Firehose Legacy dataset was used as a primary source of case information. The demographic details of the patients are given in Table 1 . The ratio of male to female was found to be 1.27: 1. The mutation count in different genes across patients was in the range of 6 to 3,181. Among the 528 samples, 504 samples had details of mutation status and copy number variation. 6 7

**Table 1:** Demographic details of HNSCC patients of the TCGA firehose legacy dataset used for analysis in the present study

Gender	Male ( *n* = 386) Female ( *n* = 142)
Mutation count	6–3,181
TP53 mutation state	TP53 mutation, *n* = 327 TP53 non-mutation, *n* = 175
Diagnosis age	19–90 y
Smoking status	Smokers: 515 Data not available: 12 Unknown: 1
Alcohol history	Yes – 352 No – 165 Data not available: 11
Neoplasm histologic grade	Grade 1: 63 Grade 2: 311 Grade 3: 125 Grade 4: 7 Grade GX: 18 Data not available: 4
Race category	White: 452 African: 48 Asian: 11 American Indian or Alaska native: 2 Data not available: 15

Abbreviations: HNSCC, head and neck squamous cell carcinoma; TCGA, The Cancer Gene Atlas.

Source: Adapted from cBioportal site.

### miRDB Database

The miRDB was used for the prediction of microRNA targets of a gene. The MirTarget was initially used for analyzing miRNA-gene target interactions from sequencing experiments. This data was then used to build the miRDB database. The miRNA targets five species namely, human, mouse, rat, dog, and chicken. There are 2.1 million gene targets regulated by 6709 miRNAs. Computational analysis and text mining were also used for functional annotation of miRNAs in human and mice. 8 9 10

### UALCAN Analysis

The expression of each of the miRNA was queried against the HNSCC dataset in the UALCAN database ( http://ualcan.path.uab.edu/cgi-bin/TCGA-survival ). The expression profile was denoted by the values transcripts per million (TPM). The unit is used for normalization of RNA-seq data. The box-whisker plots were built with the TPM values to determine the level of significance between the groups. Kaplan-Meier survival curve analysis, a multivariate process was used to deduce the survival of HNSCC patients categorized based on their expression profile. The expression profile of miRNAs was classified based on primary versus normal, *TP53* mutant versus non-mutant, normal versus *TP53* mutant, and normal versus *TP53* non-mutant. Further patterns of expression, viz., upregulation or downregulation of miRNAs in *TP53* mutant were also analyzed. 11

### EVmiRNA

Exosomes serve as carriers of information in the form of DNA, RNA, or protein. They are involved in the process of communication, hence can be an excellent source of biomarkers of diseases. Exosome synthesis is cell/tissue specific and not all extracellular vesicles derived from cells or tissues accommodate the same kind of biomarkers. Hence, the location of biomarkers remains to be the key factor to designate it as a source of potential theragnostic component for a specific disease or disorder. EVmiRNA is a user-friendly database which provides information on the exosomal location of miRNAs. The information on more than 1,000 microRNAs across 17 sources or diseases were made accessible to researchers. The EVmiRNA provides different modules for analysis such as (1) expression profiles of exosomal miRNAs from different sources, (2) details on miRNAs with differential expression, and (3) components on functional annotation of miRNA, pathway regulations, etc. 12

### ExoCarta

ExoCarta is a compendium of exosomal components derived from several cells and tissue types. The database hosts an array of biomarkers with exosomal origin and their locations or tissue type from which they are derived. The resource is made freely available for researchers to conduct experiments related to exosomes. 13 14 15

## Results

### *TP53* Mutation Analysis

**Fig. 1:**
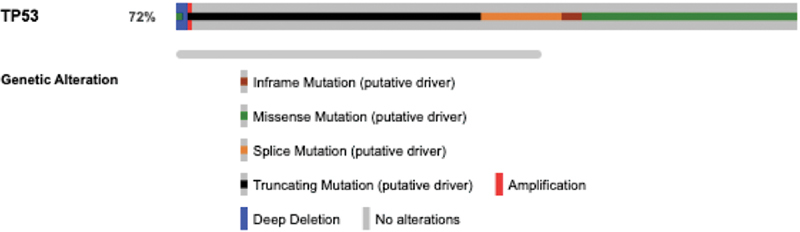
Oncoprint data demonstrating genetic alterations in TP53 gene among head and neck squamous cell carcinoma cases.

Genetic alterations in the *TP53* gene was assessed using the cBioPortal database. Among 504 HNSCC patients, 72% ( *n* = 363) presented with some kind of abnormality in the *TP53* gene ( Fig. 1 ). Aberrations included missense ( *n* = 248), splice site ( *n* = 41), inframe ( *n* = 10), truncating mutations ( *n* = 140), deep deletion ( *n* = 5), and amplication ( *n* = 2). The frequency of missense mutation was found to be high with more than 400 variations recorded in HNSCC cases. Most of these variations were identified in the *TP53* DNA binding domain with R248Q/W being identified in approximately 19 patients (data not shown). Some of the variations were found to lie within the recurrent hot spot regions of the gene.

### MicroRNA Identification

The miRDB was used to identify the potential microRNA targets of *TP53* gene. About 102 miRNAs were identified to interact with *TP53* as elucidated by a target score which ranged from 95 to 50. The distribution of miRNAs based upon the target score is as follows, between 56 and 65 it was 4, between 66 and 75 it was 4, between 76 and 85 it was 33, between 86 and 95 it was 39, and for less than 56 it was 22.

### Expression Analysis

**Fig. 2:**
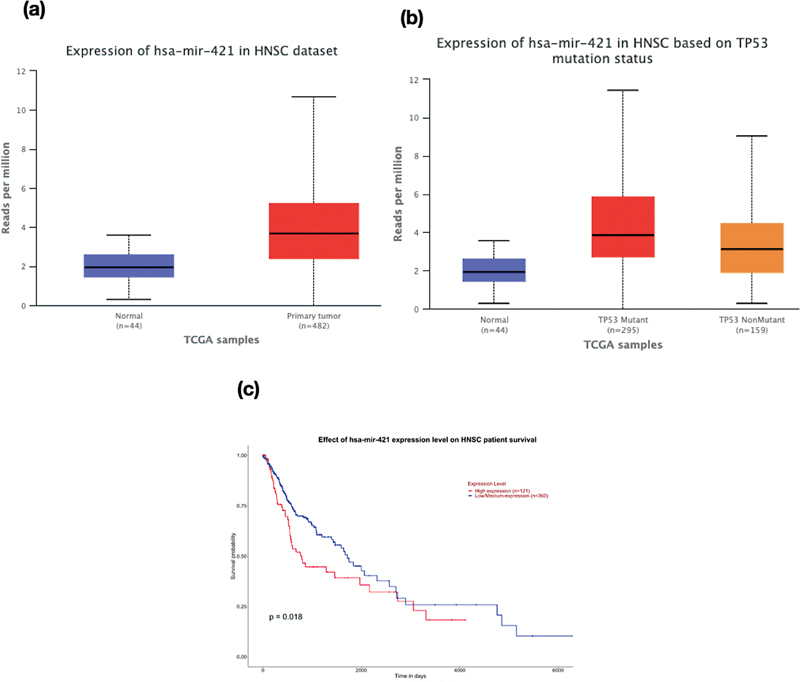
Box-Whisker plots demonstrating (a) the gene expression profile of hsa-mir-421 ( *p* value = 1.11 × 10-16) in HNSC dataset, (b) the expression profile of hsa-mir-421 based on mutation status (Normal vs TP53 mutant - *p* value = 1.62 × 10-12; TP53 mutant Vs TP53 non-mutant - *p* value = 1.15 × 10-4), (c) Kaplan-Meier survival plot showing significant difference in survival rate upon change in expression levels of hsa-mir-548f-1 ( *p* value = 0.018). A *p* value less than 0.05 is considered to be significant.

**Fig. 3:**
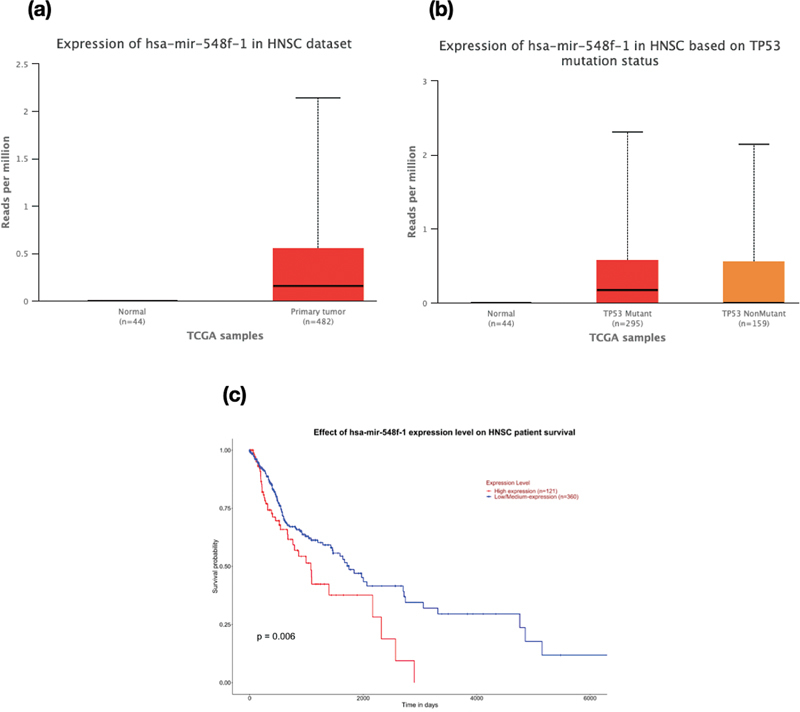
Box-Whisker plots demonstrating (a) the gene expression profile of hsa-mir-548f-1 ( *p* value = <10-12) in HNSC dataset, (b) the expression profile of hsa-mir-548f-1 based on mutation status (Normal vs TP53 mutant - *p* value = 1.81 × 10-10; TP53 mutant Vs TP53 non-mutant - *p* value = 5.28 × 10-2), (c) Kaplan-Meier survival plot showing significant difference in survival rate upon change in expression levels of hsa-mir-548f-1 ( *p* value = 0.006). A *p* value less than 0.05 is considered to be significant.

**Fig. 4:**
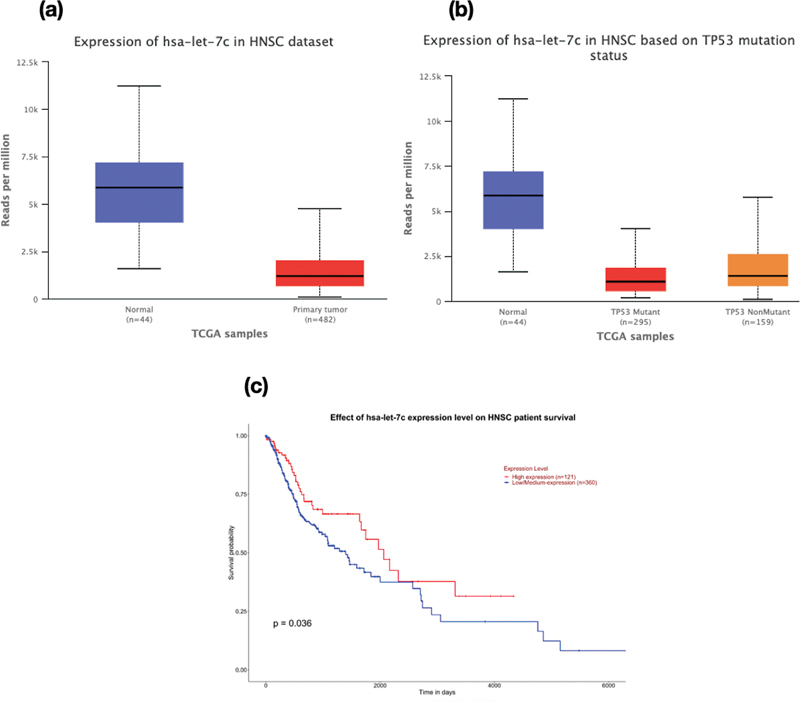
Box-Whisker plots demonstrating (a) the gene expression profile of hsa-let-7c ( *p* value = 1.11 × 10-16) in HNSC dataset, (b) the expression profile of hsa-let-7c based on mutation status (Normal vs TP53 mutant - *p* value = 1.63 × 10-12; TP53 mutant Vs TP53 non-mutant - *p* value = 2.3 × 10-3), (c) Kaplan-Meier survival plot showing significant difference in survival rate upon change in expression levels of hsa-let-7c ( *p* value = 0.036). A *p* value less than 0.05 is considered to be significant.

The miRNAs were further curated based on their expression in HNSCC dataset. The miRNAs lacking expression profile were excluded and 52 miRNAs were selected for further analysis. Among the 52 miRNAs, 12 were under-expressed and 40 were overexpressed ( Table 2 ). The observation based on survival curve analysis revealed six miRNAs showing significant difference in the number of days of survival between the low/medium expression group and high expression group of HNSCC patients. Taking together the expression values of normal versus primary tumor and survival curve, we identified three miRNAs, viz., hsa-miR-421, hsa-miR-548f-5p, and hsa-let-7c-5p. There was a marked difference observed in the expression pattern of these miRNAs across different comparison groups. The overexpression of hsa-miR-421 ( Fig. 2 ) and hsa-miR-548f-5p ( Fig. 3 ) invariably resulted in the poor survival of HNSCC patients evident from a *p* -value of 0.018 and 0.036, respectively. Similarly, the low/medium expression of hsa-let-7c-5p ( Fig. 4 ) was associated with poor survival of HNSCC patients ( *p* -value = 0.006).

### Exosomal Origin

The exosomal origin of 26 among 52 miRNAs was identified using EVmiRNA and ExoCarta database. Although microRNAs were present in other sources such as breast milk, seminal fluid, urine, etc., sources such as blood, lymph, and saliva were taken as a source of liquid biopsy. The exosomes isolated from tongue was also considered because of its close association with SCC. Oral cancer and SCC were the two cancer types defined for the present analysis. A coherent observation was made between tissue miRNA expression and exosomal miRNA expression. The two miRNAs hsa-miR-421 and hsa-let-7c-5p influencing survival of HNSCC patients were found in the blood and salivary exosomes. In addition, they were found to be present in the exosomes derived from oral cancer and SCC. The exosomal location of the 26 miRNAs were further confirmed using ExoCarta. Among the 26 miRNAs, 15 were found to be located in plasma. Majority of the miRNAs were found to be associated with colorectal cancer and melanoma ( Table 3 ).

## Discussion

The miRNAs are potent regulators of gene expression process and act by repressing translation by cleaving or hybridizing with the RNA molecule. They are 19 to 22 nucleotides in size, belonging to a class of small, non-coding RNAs. They play an important role in the process of development, cell differentiation, proliferation, and apoptosis. Accumulating evidences has suggested that these miRNAs could take up the role of oncogenes or tumor suppressors. 16 Computational predictions have demonstrated that more than 30% of the coding gene expression can be regulated or modified by miRNAs. A study by Kinoshita and team have demonstrated downregulation of miR-375 in maxillary sinus, esophageal, and hypopharyngeal SCC. The miR-375 acts as a tumor suppressor targeting several oncogenes. 17 The dysregulation of miRNAs has been implicated in numerous diseases such as metabolic, 18 autoimmune, 19 psychiatric, 20 cancer, 5 21 and many more. The encapsulation of DEM within exosomes serves as a valuable tool for diagnosing a disease by using less invasive or non-invasive methods. Targeting these molecules that interfere with their functions can bring in therapeutic value toward controlling a disease. The present study identifies potential miRNAs targeting *TP53* , a tumor suppressor gene that accumulates numerous mutations and genetic alterations in HNSCC. The differential expression and the associated survival factor revealed interesting results. Among 52 miRNAs which were differentially expressed, three were found to influence the survival of HNSCC patients with a significant *p* -value. The hsa-miR-421 and hsa-let-7c-5p were found to have an exosomal localization which makes it more stable and viable biomarker with the prognostic factor.

Numerous studies have provided substantial evidences on the functions of miRNAs in the pathogenesis of cancer. One such study aimed to determine the role of miRNA-203 in promoting tumorigenesis in hypopharyngeal squamous cell carcinoma (HPSCC). Functional analysis to identify the target genes revealed *TP63* and *B3GNT* 5 as potential targets. Although miRNA-203 was not found to play a role in invasion and metastasis, the upregulation of miRNA-203 promotes tumor growth, thereby contributing to malignancy by inhibiting tumor suppressors. 22 A similar study identified genes, miRNAs, and molecular pathways associated with HPSCC. The study returned 160 differentially expressed genes categorized based on their role in splicing, cell cycle regulation, and degradation of RNA. Around 79 miRNAs including 48 overexpressed and 31 under-expressed miRNAs were identified. 23

The *TP53* mutation has been associated with reduced survival rate in HNSCC patients. Ganci et al, investigated the miRNA targets of *TP53* in 121 HNSCC patients and correlated the outcome in 109 follow-up patients. About 58% of the patients presented with *TP53* mutation which showed a marked association with recurrence-free short survival rate. About 49 miRNAs were found to be associated with *TP53* mutation state. Of these 49, four miRNAs were related to cancer-specific survival and 12 correlated with recurrence-free survival. Three of the miRNAs were common to both groups. The study clearly demarcates 16 miRNA signatures to be associated with and selected based on survival rate in HNSCC patients. 24 The *TP53* gene is also the most commonly mutated gene in HPV-negative HNSCC. Computational analysis was performed to assess the survival rate of HPV-negative patients with mutation in comparison to those patients with TP53 wild type tumors. The outcome of the study recorded a poor survival in 203 patients who were HPV-negative. Furthermore, the levels of *SESN1, UHRF1BP11* mRNA, and miRNA-377–3p were prognostically significant in HPV-negative HNSCC patients. The results of the study could help in the stratification of patients and development of novel therapeutic strategies for HNSCC. 25 The guide and passenger strands of miRNAs are involved in the pathogenesis of cancer. A study revealed that both miR-150–5p and miR-150–3p were found to be downregulated and that they serve as anti-tumor miRNAs in HNSCC cells. There were approximately 19 gene targets for both the miRNAs which regulated the expression of *SPOCK1* gene directly. Overexpression of SPOCK1 was significantly associated with short survival rate. Thus, the study proves that the overexpression of SPOCK1 and under-expression of miRNA-150–5p/3p contributed to aggressiveness of HNSCC. 26

Coherent with the present study experimental evidences showed that miR-421 has an inherent role to play in oral cancer. The long non-coding RNA, MEG3 (maternally expressed 3) is involved in regulating the process of epithelial mesenchymal transformation (EMT) in HNSCC. The qPCR analysis performed to ascertain the expression of MEG3 in HNSCC and normal tissue at the vicinity of tumor revealed the interactions between MEG3 and miR-421, E-cadherin and miR-421. The most relevant observation acquired from the study was that there was marked downregulation of MEG3 in tumor compared with normal tissues. *In vitro* assays showed that upregulation of MEG3 prevented the proliferation, migration, and invasion of tumor cells. The interaction pathways between miR-421, MEG3, and E-cadherin regulated the process of EMT. 27 The MEG3 also contributes to stemness of oral cancer stem cells (CSCs). The downregulation of MEG3 was associated with poor prognosis. Over-expression of MEG3 controlled self-renewal and invasive abilities of CSCs. The interaction of miR-421 with MEG3 reversed the phenotype in oral CSCs driven by MEG-3 inhibition. Thus the researches postulate that overexpression of MEG-3 could serve the functions of a tumor suppressor by impeding the functions of miR-421. The present study also reports an upregulation of miR-421 with an exosomal localization which invariably resulted in poor survival of HNSCC patients. 28

Unlike other miRNAs not much information is available for hsa-let-7c-5p. A few studies have demonstrated the role of hsa-let-7c-5p in cervical squamous cell carcinoma. 29 The hsa-let-7b-5p was found to one among the seven hub miRNA associated with non-small cell lung cancer. The hsa-let-7b-5p along with two other miRNAs correlated with the survival rate of lung adenocarcinoma (LUAD) patients, portraying the prognostic value of miRNA. 30 The blood plasma profiles exhibiting miRNA expression in patients with benign hyperplasia and prostatic cancer revealed downregulation of hsa-let-7b-5p implying their functions in the development of prostatic cancer. 31

**Table 2:** List of microRNAs targeting *TP53* gene. The expression of microRNA s in different states of comparison, *p* -values for survival curve analysis

miRNA name	Normal vs. Primary tumor	Survival curve	TP53 mutant vs. non-mutant	Normal vs. TP53 mutant	Expression of microRNA in TP53 mutant	Normal vs. TP53 non-mutant
hsa-miR-3922–5p	5.5 × 10 ^−16, a^	0.14	6.5 × 10 ^−1^	5.55 × 10 ^−16, a^	Upregulated	1.72 × 10 ^−12, a^
*hsa-miR-381–3p*	2.08 × 10 ^−2, a^	0.064	9.96 × 10 ^−1^	2.07 × 10 ^−2^	Downregulated	2.08 × 10 ^−2^
*hsa-miR-6842–3p*	3.675 × 10 ^−13, a^	0.55	1.38 × 10 ^−1^	1.02 × 10 ^−9, a^	Upregulated	3.19 × 10 ^−9, a^
*hsa-miR-491–5p*	5.3362 × 10 ^−2, a^	0.92	1.28 × 10 ^−1^	3.2 × 10 ^−2^	Downregulated	1.610 ^−1^
*hsa-miR-4742–5p*	8.426 × 10 ^−4, a^	0.66	1.510 ^−1^	1.02 × 10 ^−4, a^	Upregulated	3.3 × 10 ^−3, a^
*hsa-miR-1305*	<10 ^−12, a^	0.23	5.210 ^−1^	1.62 × 10 ^−12, a^	Upregulated	4.2 × 10 ^−13, a^
*hsa-miR-548d-3p*	1.19 × 10 ^−3, a^	0.91	1.614 × 10 ^−1^	1.78 × 10 ^−2, a^	Upregulated	3.17 × 10 ^−3, a^
*hsa-miR-520b-5p*	1.33 × 10 ^−1^	0.26	3.86 × 10 ^−1^	3.78 × 10 ^−5, a^	Upregulated	2.83 × 10 ^−1^
*hsa-miR-7850–5p*	1.404 × 10 ^−10, a^	0.71	4.04 × 10 ^−1^	3.9 × 10 ^−9, a^	Upregulated	1.78 × 10 ^−5, a^
*hsa-miR-519a-2–5p*	1.903 × 10 ^−1^	0.42	3.76 × 10 ^−1^	2.16 × 10 ^−3, a^	Upregulated	3.010 ^−1^
*hsa-miR-4728–5p*	2.03 × 10 ^−2^	0.9	2.55 × 10 ^−1^	1.30 × 10 ^−1^	Upregulated	7.30 × 10 ^−2^
*hsa-miR-3652*	1.65 × 10 ^−12, a^	0.66	5.44 × 10 ^−1^	1.60 × 10 ^−10, a^	Upregulated	4.5 × 10 ^−11, a^
*hsa-miR-223–3p*	1.29 × 10 ^−1^	0.041	4.2 × 10 ^−1^	9.12 × 10 ^−2^	Upregulated	2.56 × 10 ^−1^
*hsa-miR-1294*	2.04 × 10 ^−1^	0.94	1.93 × 10 ^−1^	1.83 × 10 ^−1^	Downregulated	4.70 × 10 ^−1^
*hsa-miR-4677–3p*	<10 ^−12, a^	0.51	7.31 × 10 ^−3, a^	1.62 × 10 ^−12, a^	Upregulated	1.62 × 10 ^−12, a^
*hsa-miR-4725–5p*	2.2 × 10 ^−12, a^	0.64	1.46 × 10 ^−2, a^	3.46 × 10 ^−12, a^	Upregulated	1.75 × 10 ^−5, a^
*hsa-miR-504–5p*	1.799 × 10 ^−3, a^	0.05	6.34 × 10 ^−1^	7.38 × 10 ^−3, a^	Downregulated	1.92 × 10 ^−3, a^
*hsa-miR-4640–5p*	8.73 × 10 ^−9, a^	0.82	6.38 × 10 ^−1^	2.60 × 10 ^−8, a^	Upregulated	2.45 × 10 ^−8, a^
*hsa-miR-149–3p*	3.59 × 10 ^−3, a^	0.78	3.46 × 10 ^−1^	5.58 × 10 ^−3, a^	Downregulated	9.36 × 10 ^−2^
hsa-miR-330–3p	1.62 × 10 ^−12, a^	0.36	1.210 ^−1^	1.66 × 10 ^−12, a^	Upregulated	1.63 × 10 ^−12, a^
hsa-miR-7110–5p	5.77 × 10 ^−9, a^	0.074	1.28 × 10 ^−2, a^	2.65 × 10 ^−9, a^	Upregulated	4.36 × 10 ^−4, a^
hsa-miR-6842–5p	3.67 × 10 ^−13, a^	0.55	1.38 × 10 ^−1^	1.02 × 10 ^−9, a^	Upregulated	3.19 × 10 ^−9, a^
hsa-miR-374a-3p	6.53 × 10 ^−3, a^	0.22	8.46 × 10 ^−1^	3.26 × 10 ^−2, a^	Downregulated	3.39 × 10 ^−2, a^
hsa-miR-4529–3p	9.64 × 10 ^−5, a^	0.088	1.36 × 10 ^−1^	6.55 × 10 ^−4, a^	Upregulated	1.00 × 10 ^−6, a^
hsa-miR-6737–5p	2.28 × 10 ^−6, a^	0.19	7.010 ^−2^	1.26 × 10 ^−6, a^	Upregulated	7.82 × 10 ^−4, a^
hsa-miR-4726–5p	2.010 ^−8, a^	0.37	6.72 × 10 ^−1^	5.22 × 10 ^−7, a^	Upregulated	3.13 × 10 ^−6, a^
**hsa-miR-421	**1.110** ^−16, a^	**0.018**	**1.15 × 10** ^−4, a^	**1.62 × 10** ^−12, a^	**Upregulated**	**1.62 × 10** ^−12, a^
hsa-let-7f-5p	2.710 ^−2, a^	0.25	5.97 × 10 ^−1^	3.14 × 10 ^−2, a^	Downregulated	1.23 × 10 ^−1^
**hsa-miR-548f-5p**	**<10** ^−12, a^	**0.006**	**5.28 × 10** ^−2^	**1.810** ^−10, a^	**Upregulated**	**3.53 × 10** ^−10, a^
hsa-miR-98–5p	7.5 × 10 ^−2^	0.71	4.43 × 10 ^−4, a^	5.06 × 10 ^−3, a^	Upregulated	8.76 × 10 ^−1^
hsa-let-7b-5p	3.77 × 10 ^−2, a^	0.74	2.47 × 10 ^−1, a^	4.04 × 10 ^−2, a^	Downregulated	1.62 × 10 ^−1^
hsa-let-7 g-5p	5.60 × 10 ^−2^	0.026	4.18 × 10 ^−5, a^	2.63 × 10 ^−3^	Downregulated	7.010 ^−1^
hsa-miR-5703	1.63 × 10 ^−5, a^	0.37	4.90 × 10 ^−1^	7.75 × 10 ^−5, a^	Upregulated	3.77 × 10 ^−4, a^
hsa-miR-6731–5p	7.610 ^−10, a^	0.67	1.96 × 10 ^−2^	3.32 × 10 ^−7, a^	Upregulated	1.310 ^−9, a^
hsa-let-7a-5p	2.02 × 10 ^−4, a^	0.48	7.56 × 10 ^−3, a^	6.42 × 10 ^−5, a^	Downregulated	7.56 × 10 ^−3, a^
hsa-let-7i-5p	1.38 × 10 ^−4, a^	0.87	9.98 × 10 ^−1^	4.010 ^−4, a^	Upregulated	7.00 × 10 ^−4, a^
hsa-let-7e-5p	7.2 × 10 ^−2^	0.055	2.12 × 10 ^−1^	2.60 × 10 ^−2, a^	Upregulated	3.056 × 10 ^−1^
**hsa-let-7c-5p	**1.110** ^−16, a^	**0.036**	**2.30 × 10** ^−3, a^	**1.63 × 10** ^−12, a^	**Downregulated**	**1.62 × 10** ^−12, a^
hsa-miR-4516	2.60 × 10 ^−1^	**0.017**	4.50 × 10 ^−1^	5.38 × 10 ^−1^	Downregulated	3.2310 ^−10, a^
hsa-miR-194–3p	2.15 × 10 ^−3, a^	0.81	1.810 ^−2, a^	7.87 × 10 ^−2^	Upregulated	1.58 × 10 ^−4, a^
hsa-miR-6509–5p	3.06 × 10 ^−3, a^	0.39	9.93 × 10 ^−2^	2.00 × 10 ^−2, a^	Upregulated	4.4 × 10 ^−4, a^
hsa-miR-636	2.42 × 10 ^−14, a^	0.8	8.19 × 10 ^−2^	6.25 × 10 ^−14, a^	Upregulated	1.36 × 10 ^−7, a^
hsa-miR-939–3p	1.62 × 10 ^−12, a^	0.35	9.010 ^−02^	1.99 × 10 ^−15, a^	Upregulated	5.45 × 10 ^−10, a^
hsa-miR-3187–5p	1.46 × 10 ^−13, a^	0.11	1.22 × 10 ^−1^	1.78 × 10 ^−12, a^	Upregulated	2.03 × 10 ^−5, a^
hsa-miR-612	2.48 × 10 ^−2, a^	0.96	1.73 × 10 ^−1^	1.67 × 10 ^−1^	Upregulated	3.59 × 10 ^−2, a^
hsa-let-7d-5p	<10 ^−12, a^	0.65	2.74 × 10 ^−1^	10 ^−12, a^	Upregulated	1.62 × 10 ^−12, a^
hsa-miR-150–5p	1.97 × 10 ^−1^	0.063	2.910 ^−6, a^	4.3 × 10 ^−1^	Upregulated	1.10 × 10 ^−3^
hsa-miR-6766–5p	7.39 × 10 ^−1^	0.42	8.88 × 10 ^−1^	7.510 ^−1^	Upregulated	8.24 × 10 ^−1^
hsa-miR-21–3p	1.62 × 10 ^−12, a^	0.26	9.89 × 10 ^−2^	10 ^−12, a^	Upregulated	1.62 × 10 ^−12, a^
hsa-miR-22–3p	4.77 × 10 ^−1^	0.5	2.38 × 10 ^−4, a^	8.06 × 10 ^−1^	Upregulated	1.14 × 10 ^−1^
hsa-miR-433–5p	1.84 × 10 ^−2, a^	0.87	5.74 × 10 ^−2^	2.49 × 10 ^−2, a^	Upregulated	9.40 × 10 ^−3, a^

a, the values represent statistically significant difference in the expression profile between the groups. A *p* value less than 0.05 is considered to be significant

**Table 3:** List of TP53 targeting microRNAs with their exosome location and their exosomal expression in defined cancer cells

miRNA name	Exosome location (EVmicroRNA)	Exosomal expression in defined cancer cells	Exosome location (Exocarta)
hsa-miR-381–3p	Yes (blood)	No	Plasma
hsa-miR-6842–3p	Yes (tongue)	No	Colorectal cancer cells
hsa-miR-491–5p	Yes (tongue)	Yes (SCC)	T cells
hsa-miR-7850–5p	Yes (lymph)	No	NA
hsa-miR-223–3p	Yes (blood)	No	Colorectal, melanoma, plasma
hsa-miR-1294	Yes (blood)	No	NA
hsa-miR-4677–3p	Yes (tongue)	No	Colorectal cancer cells
hsa-miR-149–3p	Yes (saliva)	No	Plasma
hsa-miR-330–3p	Yes (blood, tongue)	No	Dendritic cells, plasma
hsa-miR-7110–5p	Yes (tongue)	Yes (SCC)	NA
hsa-miR-374a-3p	Yes (tongue)	No	Colorectal cancer cells, plasma
hsa-miR-421	Yes (blood)	Yes (oral cancer)	NA
hsa-let-7f-5p	Yes (blood, tongue)	Yes (SCC, oral cancer)	Colorectal cancer cells, plasma
hsa-miR-98–5p	Yes (blood, tongue)	Yes (SCC, oral cancer)	Colorectal cancer cells, plasma
hsa-let-7b-5p	Yes (blood, saliva, and tongue)	Yes (SCC, oral cancer)	Colorectal cancer cells, plasma
hsa-let-7 g-5p	Yes (blood, tongue)	Yes (SCC, oral cancer)	Colorectal cancer cells, plasma
hsa-let-7a-5p	Yes (blood)	Yes (SCC, oral cancer)	Colorectal cancer cells, plasma
hsa-let-7i-5p	Yes (blood, tongue)	Yes (SCC, oral cancer)	Colorectal cancer cells, plasma
hsa-let-7e-5p	Yes (blood, tongue)	Yes (SCC, oral cancer)	Colorectal cancer cells, plasma
hsa-let-7c-5p	Yes (blood, saliva)	Yes (SCC)	Colorectal cancer cells
hsa-miR-4516	Yes (blood)	Yes (oral cancer)	NA
hsa-miR-636	Yes (blood)	No	NA
hsa-let-7d-5p	Yes (blood)	Yes (SCC, oral cancer)	Colorectal cancer cells, Plasma
hsa-miR-150–5p	Yes (blood)	No	Colorectal, melanoma, plasma
hsa-miR-21–3p	Yes (blood, tongue)	Yes (SCC, oral cancer)	Colorectal cancer cells, plasma
hsa-miR-22–3p	Yes (blood, tongue)	Yes (SCC, oral cancer)	Colorectal cancer cells, plasma

Abbreviation: SCC, squamous cell carcinoma.
